# Passenger Lymphocyte Syndrome With Multiple Allo‐ and Autoantibodies Following Liver Transplantation: A Case Report

**DOI:** 10.1155/crit/8246863

**Published:** 2026-07-29

**Authors:** Ivica Marić, Tobia Gomilšek, Klara Železnik

**Affiliations:** ^1^ Slovenian Institute for Transfusion Medicine, Ljubljana, Slovenia; ^2^ Faculty of Medicine, University of Ljubljana, Ljubljana, Slovenia, uni-lj.si

## Abstract

**Background:**

Passenger lymphocyte syndrome (PLS) is a rare immune‐mediated haemolytic complication of solid organ transplantation caused by donor‐derived lymphocytes producing antibodies against recipient red blood cell (RBC) antigens. Multiple alloantibodies and concomitant autoantibody formation are uncommon, particularly in ABO‐identical liver transplantation.

**Case Presentation:**

A 56‐year‐old male underwent ABO‐identical but D‐ and Kell‐non‐identical liver transplantation. Approximately, 3 weeks posttransplantation, haemoglobin decreased to 81 g/L, and indirect antiglobulin testing identified anti‐D and anti‐E antibodies, while direct antiglobulin testing confirmed IgG‐coated RBCs. Subsequently, anti‐K alloantibodies and transient pan‐reactive IgG autoantibodies were detected, resulting in positive crossmatches and complicated transfusion support. Because most transfused RBC units before antibody detection were D+, E+ and K+, anti‐E was interpreted as probable recipient‐derived transfusion‐associated alloimmunisation, whereas anti‐D and anti‐K were consistent with donor‐derived PLS. Transfusion support was subsequently restricted to D−, E− and K− RBC units. The patient remained clinically stable despite haemoglobin decline with serologic evidence of immune‐mediated RBC sensitization consistent with PLS. During 4 years of follow‐up, anti‐D and anti‐K antibodies disappeared, while only weak residual anti‐E reactivity persisted.

**Discussion:**

The simultaneous occurrence of donor‐derived alloantibodies, probable recipient‐derived alloimmunisation and transient pan‐reactive autoantibodies suggests broader posttransplant humoral immune activation than typically observed in classical PLS. Although clinically significant autoimmune haemolytic anaemia did not develop, the coexistence of multiple antibody specificities substantially complicated serologic interpretation and transfusion management.

**Conclusion:**

This case highlights complex posttransplant humoral immune dysregulation involving donor‐derived alloantibodies, probable recipient alloimmunisation and transient autoantibody formation after ABO‐identical liver transplantation. The coexistence of multiple antibodies significantly complicated serologic testing and transfusion support.

## 1. Introduction

Passenger lymphocyte syndrome (PLS) is an immune‐mediated haemolytic complication of solid organ transplantation caused by donor‐derived lymphocytes producing antibodies against recipient red blood cell (RBC) antigens. It most commonly occurs in the setting of minor ABO incompatibility, although antibodies directed against antigens of the Rh, Kell, Kidd, Duffy, Lewis and MNS blood group systems have also been described. PLS typically develops within days to weeks after transplantation and is most frequently observed following transplantation of lymphoid tissue‐rich organs, particularly heart–lung and liver grafts [1–4].

The clinical severity of PLS ranges from mild, self‐limited haemolysis to severe or occasionally fatal anaemia. Diagnosis relies on the combination of posttransplant haemoglobin decline, biochemical evidence of haemolysis and serologic findings including positive indirect antiglobulin testing (IAT) and direct antiglobulin testing (DAT). In most cases, donor‐derived antibodies disappear spontaneously within several weeks to months as passenger lymphocytes are eliminated [1–4].

Calcineurin inhibitors such as tacrolimus predominantly suppress T‐cell activity and have been proposed to contribute to relative dysregulation of humoral immune responses, potentially facilitating B‐cell activation and antibody production [5, 6]. Although alloantibody formation is well recognised in PLS, concomitant development of multiple alloantibodies together with autoantibodies remains rare.

We report a case of ABO‐identical but D‐ and Kell‐non‐identical liver transplantation complicated by PLS with donor‐derived anti‐D and anti‐K alloantibodies, probable transfusion‐associated anti‐E alloimmunisation and transient pan‐reactive autoantibodies. Although simultaneous donor‐derived multiple alloantibodies have previously been reported, to our knowledge, no published case has described the combination of donor‐derived anti‐D and anti‐K alloantibodies, probable recipient‐derived transfusion‐associated anti‐E alloimmunisation, transient pan‐reactive autoantibodies and prolonged serologic follow‐up after ABO‐identical liver transplantation.

## 2. Case Report

In September 2018, a 56‐year‐old male with secondary biliary cirrhosis due to primary sclerosing cholangitis underwent liver transplantation at the national solid organ transplantation centre in Slovenia, with immunohaematologic support provided by our tertiary transfusion medicine institution. The recipient blood group was A, D+ and K+. Reference haemoglobin range for adult males at our institution is 135–175 g/L.

Routine pretransplant immunohaematologic testing performed 5 months (April) before transplantation, including ABO confirmation, IAT, IAT using papain‐treated reagent RBCs and DAT, showed no evidence of irregular RBC antibodies. Repeat IAT performed 2 months (July) before transplantation remained negative.

The patient received an ABO‐identical liver graft from a donor with D− and K− phenotype. Posttransplant immunosuppressive therapy included tacrolimus and mycophenolate mofetil according to institutional transplantation protocols. During transplantation, the patient received four units of A, D+ RBCs and six units of A plasma. Six days after transplantation, repeat IAT remained negative.

Approximately 3 weeks posttransplantation (October), haemoglobin decreased to 81 g/L, and IAT became positive, identifying anti‐D and anti‐E alloantibodies. DAT was positive for IgG‐coated RBCs, while eluate testing demonstrated anti‐D reactivity. Molecular typing confirmed the recipient RH phenotype as D+, C+E−c−e+. Two A, D−, E− RBC units were transfused.

Approximately 2 months (November) after transplantation, haemoglobin further decreased to 68 g/L. Additional serologic testing demonstrated newly detectable anti‐K alloantibodies together with pan‐reactive IgG autoantibodies, resulting in positive crossmatches with donor RBCs. A second eluate study demonstrated pan‐reactive IgG reactivity. Subsequent targeted alloadsorption studies using selected RBC phenotypes confirmed the previously identified alloantibody specificities and excluded additional underlying alloantibodies. Extended RBC genotyping showed K+k+, Jk (a+b+), Fy (a+b−), M+N− and S−s+ phenotype. Two compatible A, D−, E−, K− RBC units were transfused, increasing posttransfusion haemoglobin to 86 g/L.

Following adjustment of transfusion support, the patient remained clinically stable despite haemoglobin decline with serologic evidence of immune‐mediated RBC sensitization. Haemoglobin subsequently stabilised between 84 and 93 g/L, and the patient was discharged approximately 3 months (December) after transplantation. No severe haemolytic crisis, graft dysfunction or transfusion‐related adverse event occurred during follow‐up.

Overall, the patient received 18 RBC units (~5000 mL), including 5 units before transplantation, 4 units during liver transplantation and 9 units during the posttransplant period. Before the onset of PLS, most transfused RBC units were D+, E+ and K+, whereas after antibody identification, transfusion support was restricted to donor‐ and recipient‐compatible A, D−, E−, K− RBC units. At 4‐year follow‐up, both IAT and DAT were negative. Only weak anti‐E reactivity persisted using papain‐treated reagent RBCs, suggesting residual low‐level sensitisation.

All serologic investigations were performed in the transfusion medicine laboratory using the column agglutination technique (CAT) with commercially available Bio‐Rad reagents, test cells and gel card systems (Bio‐Rad, Cressier, Switzerland), according to the manufacturer′s instructions. Additional investigations included two eluate studies, alloadsorption procedures and extended RBC genotyping according to routine laboratory protocols. Biochemical markers of haemolysis, including lactate dehydrogenase, haptoglobin, indirect bilirubin and reticulocyte count, were not consistently available in the accessible clinical documentation because of the retrospective nature of this case report.

**Figure 1:**
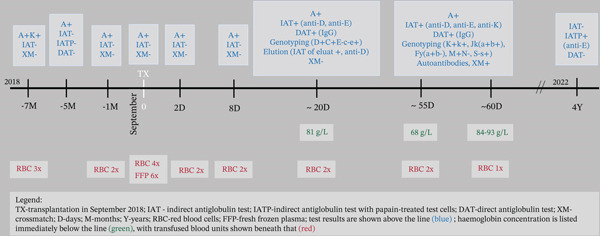
Timeline of serologic findings, haemoglobin values and transfusion support following ABO‐identical liver transplantation.

The precise timeline of serologic findings, transfusions and haemoglobin changes is presented in Figure 1.

## 3. Discussion

PLS is a recognised complication of solid organ transplantation resulting from donor‐derived lymphocytes producing antibodies against recipient RBC antigens. Unlike ABO incompatibility, D and Kell antigen mismatches have no direct immunological significance for liver graft survival but remain clinically relevant because they may lead to PLS and complicate transfusion support. In our patient, the timing of antibody appearance approximately 3 weeks after transplantation, combined with donor–recipient D and Kell incompatibility, positive DAT findings and the transient nature of anti‐D and anti‐K antibodies, strongly supported the diagnosis of PLS [1–4]. Most reported PLS cases involve a single antibody specificity, predominantly ABO isoagglutinins, whereas non‐ABO antibodies are substantially less common. Among non‐ABO antibodies, anti‐D, anti‐E and anti‐c are the most frequently reported Rh specificities, while anti‐K‐mediated PLS has only occasionally been described [1–4]. Simultaneous detection of multiple alloantibodies remains rare and is largely limited to isolated case reports. The simultaneous detection of multiple antibody specificities in our patient therefore suggested broader posttransplant humoral immune activation rather than isolated donor‐derived alloimmunisation alone.

Anti‐D and anti‐K antibodies were most consistent with donor‐derived alloantibody production, given the donor D−/K− and recipient D+/K+ antigen mismatch. In contrast, the origin of anti‐E antibodies was less certain. Because the recipient was E‐negative and had received multiple E‐positive RBC transfusions before antibody detection, anti‐E most likely represented recipient‐derived transfusion‐associated alloimmunisation rather than classical donor‐derived PLS. Although anti‐E was first detected approximately 3 weeks after transplantation, the recipient had also received RBC transfusions seven and 1 month before transplantation. These earlier transfusions may have resulted in prior sensitization with subsequent anamnestic antibody production following additional peritransplant exposure to E‐positive RBC units. The temporal overlap of donor‐derived alloantibodies and recipient alloimmunisation complicated the serologic interpretation.

Compared with previously published reports, our case is notable for the coexistence of donor‐derived alloantibodies, probable recipient‐derived alloimmunisation and transient pan‐reactive autoantibody formation during the same posttransplant period. Although Monfort et al. described simultaneous donor‐derived multiple alloantibodies following liver transplantation, to our knowledge, we were unable to identify a previously published report describing this specific combination of donor‐derived anti‐D and anti‐K alloantibodies, probable recipient‐derived transfusion‐associated anti‐E alloimmunisation and transient pan‐reactive autoantibody formation in a single liver transplant recipient [7]. This combination supports the possibility of broader polyclonal B‐cell activation in the early posttransplant setting [7, 8]. While positive DAT findings and nonspecific autoantibody reactivity have occasionally been described in transplant recipients, clinically significant autoimmune haemolytic anaemia (AIHA) remains uncommon under immunosuppressive therapy [9].

Calcineurin inhibitors such as tacrolimus primarily inhibit T‐cell activation through suppression of interleukin‐2 transcription and downstream T‐cell proliferation. Because B‐cell activity is less directly suppressed, impaired regulatory T‐cell control may permit dysregulated humoral immune responses, including autoreactive and alloimmune antibody production [5, 6, 10]. In the posttransplant setting, additional immune stimulation from donor passenger lymphocytes, transfusion‐related antigen exposure, inflammatory cytokine activation and tissue injury may further contribute to transient polyclonal B‐cell activation [11, 12]. Although the exact mechanism cannot be established in the present case, these factors, together with tacrolimus‐based immunosuppression, may have contributed to the simultaneous development of multiple alloantibodies and transient pan‐reactive autoantibodies. Similar observations have been described in rare reports of posttransplant immune dysregulation associated with tacrolimus‐based immunosuppression [5, 6]. In the absence of tacrolimus dosing and therapeutic drug monitoring data, however, this proposed mechanism should be considered hypothesis‐generating rather than evidence of a causal relationship. Nevertheless, despite DAT positivity and the presence of pan‐reactive autoantibodies, the patient did not fulfil criteria for clinically significant AIHA [9]. The patient remained clinically stable, and the observed haemoglobin decline with serologic evidence of immune‐mediated RBC sensitization was temporally consistent with concurrent PLS‐related alloimmune RBC destruction, without evidence of progressive autoimmune haemolysis.

The clinical significance of this case therefore extends beyond the haemolysis itself. The coexistence of multiple alloantibodies and autoantibodies substantially complicated transfusion support and pretransfusion compatibility testing, resulting in positive crossmatches and the need for extended antigen matching with donor‐ and recipient‐compatible D−, E− and K− RBC units. Early recognition of PLS and close collaboration between transplantation and transfusion medicine teams were essential to avoid further haemolytic exposure and ensure compatible transfusion support.

At long‐term follow‐up, anti‐D and anti‐K antibodies became undetectable, consistent with the transient nature of donor‐derived passenger lymphocyte activity. Persistent weak anti‐E reactivity likely reflected residual recipient alloimmunisation following prior antigen exposure.

## 4. Conclusions

This case highlights the complexity of posttransplant humoral immune interactions, in which donor‐derived passenger lymphocytes, transfusion‐related antigen exposure and immunosuppression‐associated immune dysregulation may simultaneously contribute to allo‐ and autoantibody formation. The peculiarity of this case lies in the simultaneous presence of donor‐derived anti‐D and anti‐K alloantibodies, probable recipient‐derived anti‐E alloimmunisation, transient pan‐reactive autoantibodies and long‐term serologic follow‐up after ABO‐identical liver transplantation. To our knowledge, this combination has not previously been reported in the literature. In addition to haemolysis, these immune phenomena substantially complicated serologic testing and transfusion support. Early recognition of PLS and close multidisciplinary collaboration between transplant and transfusion medicine teams remain essential for optimal patient management and prevention of antibody‐mediated complications.

## Funding

No funding was received for this manuscript.

## Consent

Written informed consent was obtained from the patient for the publication of this case report and any accompanying images.

## Conflicts of Interest

The authors declare no conflicts of interest.

## Data Availability

The data that support the findings of this study are available on request from the corresponding author. The data are not publicly available due to privacy or ethical restrictions.
